# Perceptions of health data commodification in AI-driven healthcare systems in Saudi Arabia

**DOI:** 10.3389/frai.2025.1559302

**Published:** 2025-11-04

**Authors:** Marran Al Qwaid

**Affiliations:** Department of Computer Science, College of Computing and Information Technology, Shaqra University, Shaqra, Saudi Arabia

**Keywords:** health data commodification, AI, digital healthcare, AI-driven healthcare, trust in AI systems

## Abstract

**Introduction:**

Artificial Intelligence (AI) is transforming healthcare service delivery through predictive analytics, precision medicine, and advanced diagnostics. However, the commodification of health data introduces complex ethical and social challenges related to privacy, ownership, and consent. This study explores perceptions of health data commodification within AI-driven healthcare systems, focusing on Saudi Arabia’s rapidly evolving digital healthcare landscape.

**Methods:**

A mixed-methods approach was employed, combining quantitative surveys and in-depth qualitative interviews. The study included 42 patients, 8 healthcare professionals, 3 insurance representatives, and 4 AI experts. Data were collected across three main themes: data privacy, perceived benefits of AI, and attitudes toward data commodification. Quantitative data were analyzed descriptively, while qualitative responses were examined thematically.

**Results:**

Findings reveal that 61.9% of patients consider health data a form of personal property, while 59.5% feel they have limited control over how their data are used. A significant trust deficit was observed, with 50% expressing low confidence in AI systems’ ability to protect privacy, particularly among older participants. Financial incentives strongly influenced willingness to share data, with 81% agreeing to share their data if compensated. Furthermore, 64.3% supported the sale of anonymized data by healthcare providers to technology companies, provided adequate safeguards are in place.

**Discussion:**

These insights underscore the urgent need for robust regulatory frameworks emphasizing informed consent, transparency, and ethical governance in AI healthcare systems. The study highlights the importance of patient-centered policies, equitable compensation mechanisms, and enhanced training and awareness programs to build public trust and ensure responsible AI adoption. By addressing these ethical and governance challenges, policymakers can align technological innovation with equity, privacy, and the principles of ethical healthcare delivery.

## 1 Introduction

Artificial Intelligence (AI) has emerged as a tool to facilitate improvements in healthcare, leading to a drastic transformation in healthcare service delivery. From diagnostics and patient monitoring to data-driven health management, healthcare has been redefined in this era (Silcox et al., 2024). Central to this transformation is the use of extensive health data, which has led to enhancements in predictive analysis at an unprecedented scale and has enabled more effective assessments of patient outcomes (Abnousi et al., 2019). Beyond its clinical value, health data is increasingly recognized as an economic asset. This data has been leveraged by policy makers in making evidence-based decisions utilising insights about demographic characteristics and population health. This has been especially useful in the identification of vulnerable populations aiding in the development of targeted interventions (Basu et al., 2020). The surge in digital health data production, facilitated by technologies like electronic health records (EHR) and wearable devices (e.g., smartwatches and glucose monitors), has further enhanced AI algorithms’ precision in predictive analytics (Dickens, 2020; Sharon, 2018).

As Lupton (2014) notes, even personal health narratives shared online are now commodified within the digital health economy, blurring distinctions between personal health information and marketable data assets. This trend highlights health data’s vulnerability to commodification posing significant ethical and social challenges. These challenges are accompanied by a heightened concern over increased costs, data ownership rights, and limited transparency in data usage (Khullar et al., 2022; Krüger and Wilson, 2023).

Extant literature has highlighted these ethical concerns, with issues ranging from diminished data privacy, informed consent, individual autonomy, and the potential exploitation of sensitive health information (Kim et al., 2019; Lupton, 2014; Ostherr, 2022; Vellido, 2019). Bridge et al. (2021) further elucidate the arising risk of access of personally identifiable information among data brokers, which may be misused towards advertising and other purposes. Furthermore, as highlighted by Gallese (2024), there are pressing concerns arising between health data commodification and citizens’ rights in European Union’s digital market, emphasizing the need for regulatory measures and frameworks (Crain, 2018). However, as pointed out by Siala and Wang (2022), much of the research offers a Western viewpoint as studies have primarily arisen from West Europe and North America and do not provide comprehensive insights from other parts of the world.

Even though the research has aimed to examine and evaluate the challenges and perceptions of medical students and practitioners with respect to the utilisation of AI in healthcare (Al-Ani et al., 2024), research on understanding patients’ perceptions of the utilisation of their health data as an economic asset, especially within AI-driven healthcare ecosystems is limited as highlighted by a systematic review on stakeholder perspectives wherein over 16,000 articles were assessed (Kuo et al., 2024).

Given the highly sensitive nature of health data, it is essential to explore perspectives from patients on data commodification, its potential as a privacy infringement, or a necessary trade-off for technological advancements. From a patient’s viewpoint, this practice may be seen as a violation of privacy, however, commodified health data could lead to radical improvement in healthcare. By leveraging large datasets, breakthroughs could be achieved in precision medicine, diagnostics, disease prediction, and global health initiatives.

This study addresses the gap in patient perspectives by examining individuals’ perceptions of their health data being commodified in AI health systems. It provides both quantitative and qualitative insights into their attitudes, concerns, and expectations surrounding the commodification of health data. In order to get a comprehensive understanding of perceptions of health data commodification in AI-driven healthcare, the study includes a wide spectrum of stakeholders other than just patients. These stakeholders include healthcare workers, health insurance players, and AI/health tech professionals. From the AI point of view, these stakeholders are all key players: a healthcare professional would be the voice of ethics and practical clinical applicability of AI tools; a health insurance player would provide inputs on economic and regulatory considerations around data commodification; and the AI/techie would discuss the technical feasibility, potential algorithmic bias, and broader implications of using patient data in AI. Capture these perspectives such that the study contextualizes patient experiences within the operational, ethical, and technological realities of AI in healthcare as well. Such a multi-stakeholder approach ensures that findings are actionable, relevant, and are a big step toward making responsible AI a reality within the Saudi healthcare ecosystem. Specifically, the study hypothesizes that participants’ perceptions may vary based on their trust in AI-driven healthcare systems, expectations of compensation, and views on privacy and control over data use. These hypotheses have been tested through a mixed-method approach, including surveys, and qualitative responses, to capture the perspectives of both healthcare providers and patient groups. This study gathered data and insights from patients, healthcare professionals, health insurance representatives, and AI/healthcare technology experts. A diverse stakeholder perspective was targeted as this is essential to ensure that the ethical, clinical, economic, and technical dimensions of AI-driven data practices were comprehensively addressed.

Healthcare professionals’ were included due to their critical role in informing development and utilisation of AI tools within clinical settings. Their perspectives help to assess whether AI applications align with the realities of medical practice and uphold ethical standards of care. Health insurance representatives’ views were engaged to provide insights into the economic implications of AI-driven data usage, particularly in terms of data commodification and its implications for reimbursement models and regulatory compliance. The insights from AI/healthcare technology experts are paramount to understand the technical implications and potential risks, such as algorithmic bias, and exploring the broader implications of using patient data in commercial and non-clinical contexts.

Notably, non-patients outside of these defined stakeholder categories were not included in the study, as the focus was on individuals directly involved in or affected by the development, implementation, and governance of AI in healthcare. This targeted approach ensured that the data collected was both relevant and actionable for informing responsible AI integration in the healthcare domain. This article aims to explore and examine individuals’ perceptions of their health data being commodified in AI health systems, particularly in the context of Saudi Arabia. It makes a meaningful contribution to the evolving landscape of artificial intelligence in healthcare by examining the socio-ethical implications of data commodification through the lens of key stakeholders. It provides novel insights into user trust in AI systems by uncovering how perceptions of data ownership, transparency, and consent influence willingness to engage with AI tools in clinical settings. Furthermore, the study identifies both barriers and facilitators to AI adoption. Insights from this study will not only inform AI developers, policymakers, and healthcare providers on data management perspectives within the Saudi healthcare system but also contribute to the growing global discourse on responsible data governance in AI health ecosystems. Saudi Arabia’s case offers a unique lens through which to examine these dynamics, underscoring broader implications for AI-driven health systems worldwide.

## 2 Methods

### 2.1 Study design and participants

This study employed a concurrent mixed-methods approach to explore perceptions of health data commodification within AI-driven healthcare systems. This method enabled the collection of quantitative and qualitative data towards a robust data collection framework as well as allowed capturing of qualitative insights (Goertzen, 2008). Data collection included quantitative and qualitative surveys conducted with 42 patients, alongside in-depth interviews with 8 healthcare professionals, 3 health insurance representatives, and 4 individuals with expertise in AI and healthcare technology. Data collection took place from 22 October to 20 November 2024. Participants were recruited to provide a broad perspective on the topic, with a focus on diverse roles in healthcare and data ecosystems.

### 2.2 Data collection tools and procedures

Quantitative data were collected using structured questionnaires hosted on Google Forms, designed to capture patients’ attitudes and perceptions regarding health data commodification. The survey comprised 20 questions rated on a 5-point Likert scale and 10 open-ended qualitative questions exploring personal views and concerns on the commercialisation and commodification of health data.

Qualitative data were gathered through in-person interviews and structured discussions. Interview guides were tailored to each participant group, addressing their specific roles and experiences. For healthcare professionals and insurance representatives, the discussions focused on ethical considerations and policy frameworks, while with AI experts the discussions included a focus on technological and commercial implications of health data use. Each interview lasted 30–45 min.

The study reached data saturation with the selected sample size, which included participants from four key stakeholder groups: patients, healthcare professionals, health insurance representatives, and AI/healthcare technology experts. Participants were recruited through convenience and purposive sampling. Invitations were sent through online announcements and organizational networks. Sample saturation was confirmed when interviews and surveys produced no new codes or themes related to the research questions. This showed that we had enough coverage of perspectives from different stakeholder groups. Saturation was determined when consecutive interviews and focus group discussions yielded no substantially new codes, themes, or perspectives relevant to the study’s core research questions. The recurrence of key concepts—such as trust in AI systems, concerns over data ownership and commodification, and context-specific ethical considerations—indicated that the dataset was sufficiently rich and comprehensive to support robust thematic analysis. The diversity within and across stakeholder groups further contributed to the depth and breadth of insights, reinforcing the adequacy of the sample size for the study’s qualitative aims.

### 2.3 Survey instruments

The patient questionnaire included demographic questions and a series of statements evaluating perceptions on privacy, control, and economic aspects of health data commodification. Open-ended questions explored deeper insights into participants’ beliefs, concerns, and expectations. Interview guides for professionals covered broader systemic perspectives, ethical dilemmas, and practical implications of health data management in AI systems. These instruments were refined through pilot testing with five individuals to ensure clarity and relevance. The design of data collection instruments emphasized participants’ understanding and concerns regarding the use of their health data in AI algorithms. Survey and interview questions elicited views concerning data privacy implications, algorithmic biases, and perceptions of data commodification in AI-assisted healthcare systems. Thus, the answers collected reflected general attitudes on the use of health data but also specific views on ethical, technical, and practical challenges posed by AI in healthcare. The explicit probe of such AI issues further enhances the study’s attractiveness to researchers, policymakers, and practitioners in the context of developing a responsible and trustworthy AI system in Saudi healthcare.

### 2.4 Recruitment and sampling

Participants were recruited using convenience and purposive sampling. Patients were approached via online announcements, while professionals were identified through organizational networks and direct invitations. Participation was voluntary, with informed consent obtained electronically or verbally before data collection.

### 2.5 Data storage and protection

No identifiable information was collected for the online survey mode or for in-person discussions.

### 2.6 Data analysis

Data was analysed for participants who gave informed consent, were 18 years or older, and achieved 100% progress in the questionnaire. Quantitative survey responses were analyzed using descriptive and inferential statistics to identify patterns in patients’ attitudes. Qualitative data from open-ended questions and interviews were transcribed and analyzed thematically, using coding frameworks to identify recurring themes and insights across participant groups, following the approach outlined by Braun et al. (2022). Qualitative data were coded separately by two researchers. They compared their codes to check for agreement. Any discrepancies were discussed until they reached a consensus, resulting in a 92% agreement rate. This process helped ensure reliable and consistent identification of themes. Members of the research team reviewed interview transcripts and participant responses in order to refine coding and develop themes.

### 2.7 Ethical considerations

The study adhered to ethical guidelines, with all participants providing informed consent. Data were anonymized to protect participants’ identities, and secure storage ensured confidentiality. Ethical approval was obtained from the Research Ethics Committee (REC) at the College of Computing and Information Technology at Shaqra University with the Ethics Application number 0121102024. To improve patient autonomy, we considered dynamic consent models. This approach lets participants review, change, or withdraw their consent over time. It gives them more flexibility and control over how their health data is used.

This exploration is particularly timely in light of Saudi Arabia’s evolving healthcare landscape, where digitalization and public-private partnerships are increasingly shaping healthcare delivery. In 2023, Saudi Arabia invested over $50 billion in various digital health initiatives (El-Shaeri, 2023), including a partnership with Orion Health to create the world’s largest health information exchange—leveraging data from over 32 million citizens to design targeted healthcare interventions (Ang, 2022). Additionally, under its National AI Strategy 2031, Saudi Arabia has identified healthcare as a priority sector for AI innovation (Saudi Data and Artificial Intelligence Authority, n.d.).

## 3 Results

Survey responses were collected from 42 individuals (33.33% male, 59.52% female and 7.14% responded “prefer not to say”), evenly distributed across genders and a range of age groups (18–65 years). Most respondents had a bachelor’s degree or equivalent. Key demographic variables are presented in Table 1. Further, the analysis of the qualitative responses revealed several themes such as concerns over data commodification, requirements of necessary safeguards, implications for healthcare quality and accessibility, and the influence of financial incentives.

**Table 1:** Socio-demographic characteristics.

Variable	Category	Count	Percentage (%)
Gender	Male	24	33.33
	Female	13	59.52
	Prefer not to say	5	7.14
Age group	18–25 years	8	19.05
	26–34 years	11	26.19
	35–44 years	14	33.33
	45 years and above	9	21.43
Education level	High school or diploma	8	19.05
	Bachelor’s degree or equivalent	25	59.52
	Postgraduate	9	21.43

### 3.1 Attitudes towards data ownership and privacy

**Figure 1:**
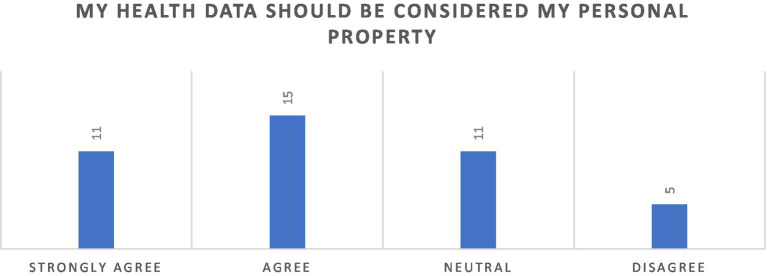
Perspectives on data ownership among study participants.

A majority of survey respondents (61.90%) agreed with the statement that health data should be regarded as their personal property, while only a minority (11.90%) expressed disagreement, indicating that they do not perceive their health data as a personal asset (Figure 1). A higher proportion of males, 7 out of 24, while 29.17% indicated a stronger agreement with the statement. Agreement was consistent across all age groups, with older respondents (45 years and above) displaying the strongest agreement 100% agreeing or strongly agreeing, with no participants reporting disagreement. The responses reflect widespread consensus on the personal ownership of health data across demographic categories.

**Figure 2:**
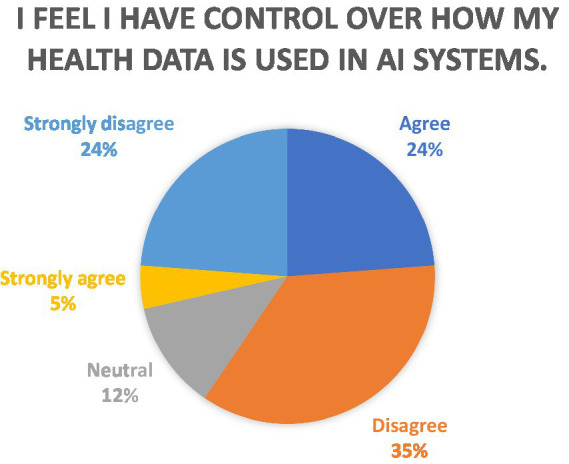
Perceptions on control over data use by AI systems.

Furthermore, the study also explored the perceptions on control over the use of health data by AI systems as seen in Figure 2. The data reveals that a significant majority of respondents expressed disagreement with the statement, “I feel I have control over how my health data is used in AI systems.” A majority of respondents expressed disagreement, highlighting a widespread perception of lack of control over health data usage in AI systems. This demonstrates a widespread perception of a lack of control over health data usage in AI systems. Among the diverse age groups, the highest level of disagreement was observed among those in the later age cohorts, wherein those above the age of 45 years, a majority (77.8%, or 7 respondents) strongly disagreed or disagreed, reflecting a pronounced sense of a lack of control.

**Figure 3:**
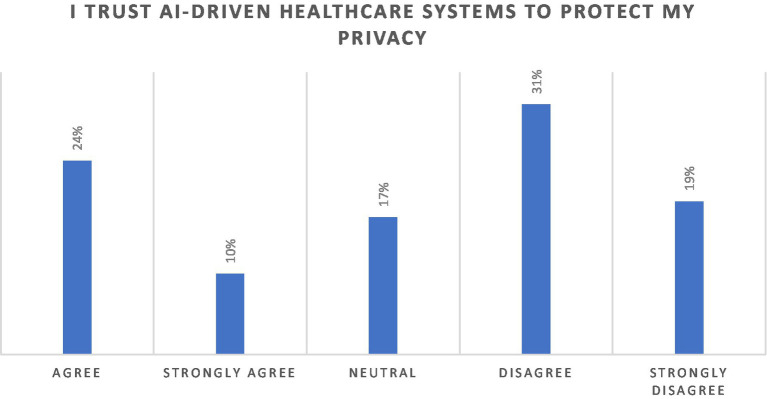
Perspectives on the statement “I trust AI-driven healthcare systems to protect my privacy”.

The data also presents perspectives on trust and privacy, in Figure 3, wherein half of the participants (50%) disagreed and strongly disagreed with “I trust AI-driven healthcare systems to protect my privacy”, indicating a deep trust deficit. Age-based trends were also evident; older respondents (45 years and above) exhibited higher levels of distrust or strong disagreement, possibly reflecting heightened scepticism toward new technologies. Conversely, younger respondents (18–24 years) were relatively more neutral, indicating either a lack of experience with such systems or an open perspective. The data underscores a polarized perspective on AI-driven healthcare systems, with significant scepticism coexisting alongside moderate levels of trust.

Qualitative responses from the participants provided some keys insights on concerns about trust, control, scepticism, and accountability in the context of health data ownership. Trust in healthcare providers and AI systems was a recurring concern. One participant commented, “*It’s unsettling, and I feel that I have become another source of income, not a patient.*” Another shared, “*It affects the trust since I may be treated differently based on the commercial value of my data.*” Scepticism about corporate motives was widespread, with participants noting that “*Commodification of health data may place corporate interests above patient privacy and consent.*” The demand for control over personal health data was evident. Participants emphasized the importance of informed consent and the ability to revoke it at any time. One respondent explained, “*I’d allow it if I could opt-in voluntarily and have the option to change my mind at any given time.*” The call for accountability was equally strong, with participants stressing the necessity of transparency: “*If the sale of health data results in improvement to the healthcare system, so be it, but transparency is of essence.*” AI and healthcare technology experts emphasized the critical importance of robust technical measures and patient-centered consent processes. Data encryption and advanced technologies were repeatedly highlighted as essential safeguards. One expert stated, *“Strong encryption and secure access controls will be paramount to protect patient data”*.

Perspectives from healthcare professionals shows that privacy and informed consent could be the cornerstone for building trust and would be central to the ethical use of health data in AI-driven healthcare systems. Doctors, nurses, and administrators underscored the need for informed consent and transparency in data handling. A general practitioner worried about the perception of patients as “sources of income” rather than individuals, stating that a lack of transparency could erode trust. Similarly, an emergency room nurse emphasized that patients must have clear choices and control over how their data is shared, with the ability to withdraw consent at any time. The oncologist further stressed that informed consent must be an ongoing process, especially in research-heavy fields like oncology. Across roles, there was consensus that patients should always remain in control of their data, and professionals must advocate for their rights.

### 3.2 Perceptions of health data commodification

**Figure 4:**
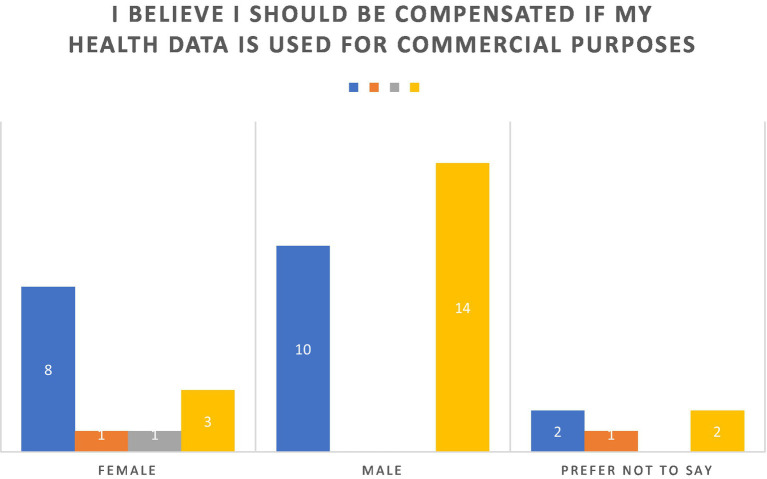
Perspectives on compensation for data commodification.

There is a strong consensus among survey respondents towards receiving compensation for utilization of health data for commercial purposes as seen in Figure 4. Almost all participants (92.86%) expressed agreement. Respondents with higher educational qualifications, such as graduate degrees, showed particularly strong alignment, as 100% of this group either agreed or strongly agreed, with a notable majority strongly agreeing. Similarly, individuals with high school diplomas and bachelor’s degrees exhibited near-unanimous support. Younger respondents (18–24 years) and those in the middle-aged categories (25–44 years) mirrored this consensus, with no recorded disagreement and only minimal neutrality. Interestingly, the older cohort (45 years and above) demonstrated unanimous support, suggesting that perceptions of compensation for health data usage may be informed by lived experiences or heightened sensitivity to privacy concerns. Gender-based trends further underline the universality of this perspective, as males, females, and those preferring not to disclose their gender overwhelmingly supported the notion of compensation. Disagreement was virtually negligible (4.76%), with only two respondents voicing opposition. These findings underscore a broad recognition of the commercial value of personal health data and an expectation of equitable compensation for its use, reflecting evolving societal attitudes toward data privacy and ownership in an increasingly digital and data-driven economy.

**Figure 5:**
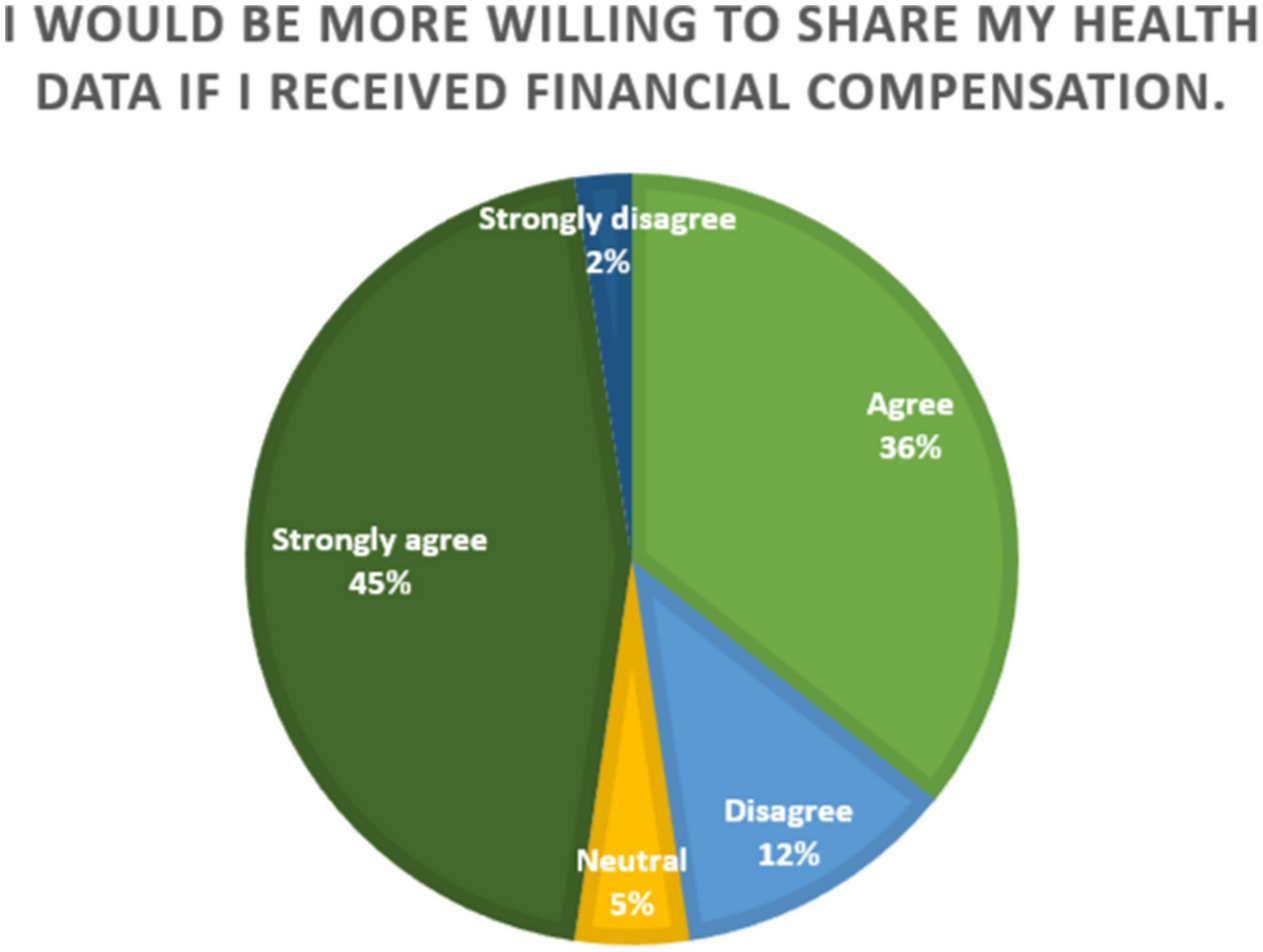
Perspectives on willingness to share data.

Further, Figure 5 illustrates the distribution of responses to the statement, “I would be more willing to share my health data if I received financial compensation.” The data reveals a strong inclination toward agreement, with 81% of respondents either agreeing (36%) or strongly agreeing (45%). A small minority expressed disagreement, with 12% disagreeing and only 2% strongly disagreeing. The agreement of the majority of the respondents suggests that financial compensation would be a key motivator towards willingness to share data. This implies that individuals perceive their health data as a valuable personal asset and expect equitable returns for its use, particularly in commercial or research contexts. However, 14% of the participants disagreed with the sentiment, indicating that concerns about data sharing may stem from factors other than compensation, such as privacy risks or data misuse. Since a very small percentage of participants remained neutral on the issue (5%), which reflects the importance of financial compensation as a clear determinant in the willingness to engage in health data sharing.

Furthermore, the study also explored the perceptions of the participants to the following statement “Healthcare providers should be allowed to sell anonymized patient data to tech companies” wherein a majority (64.29%) expressed agreement or strong agreement, reflecting a general acceptance of the practice of selling anonymized data, likely due to its perceived benefits for research, innovation, and technological development. However, a notable minority (23.81%) expressed disagreement or strong disagreement, signaling concerns about privacy, ethics, or trust in healthcare providers and tech companies to handle data responsibly.

**Figure 6:**
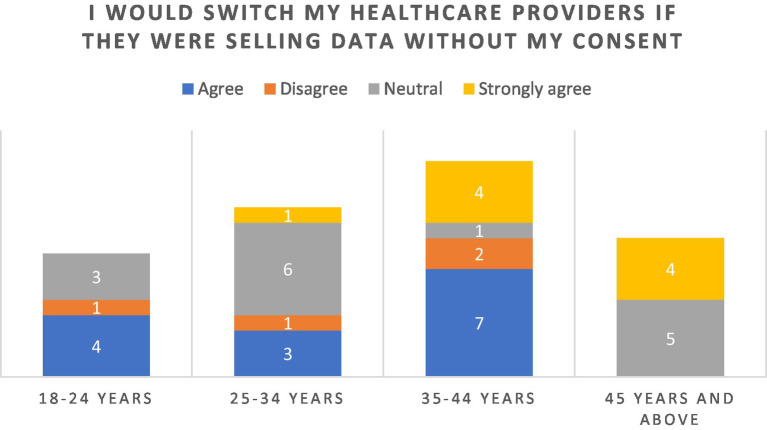
Perception of selling of data without consent.

In response to the statement “I would switch healthcare providers if I found out they were selling my data without my consent”, majority (54.76%) of the respondents reported “agreeing” or “strongly agreeing” (Figure 6). A particularly strong stance was seen among the older cohorts of ages 35–44 and those over the age of 45 years. Responses were broadly consistent across genders, with both males and females expressing high levels of agreement and strong agreement.

Responses to health data commodification were diverse, reflecting both conditional acceptance and ethical apprehensions. A widely shared sentiment was, “*I do not mind if my health data is sold, so long as it’s anonymous and I’m aware of how it’s being used.*” Anonymization and explicit consent were emphasized as non-negotiable safeguards. Concerns about misuse dominated many discussions. Participants feared “*the potential for misuse by insurance companies or employers*” and others worried that the motive would be profit-driven: “*Commodification of health data may place corporate interests above patient privacy and consent*”. Economic disparities or inequity in access to rural and underserved populations were another focus, as participants observed that commodification might “*widen the gap between those who can afford premium services and those who cannot*”.

Despite these reservations, some participants acknowledged potential benefits. “*If it leads to cheaper or better healthcare, I might be more open to the idea*,” remarked one respondent. Financial incentives also shaped perceptions, with another noting, “*If I could profit from my data being sold, I’d feel better about it.*” Healthcare technology experts also emphasized on the potential of AI in healthcare as stated “*Commodification of health data will catalyze AI development by having larger and more diverse training datasets, better diagnostics, and personalized treatments*”.

Further, healthcare providers warned against the ethical concerns of data commodification. A healthcare administrator acknowledged the potential of data commodification to allocate resources effectively through AI, but warned against prioritizing profit over patient outcomes. Similarly, a cardiologist emphasized that commodification could conflict with patient-centered care, risking reduced quality due to profit-driven motives. Ethical concerns were pronounced, with a critical care nurse warning about biases in AI systems trained on commercialized data, potentially leading to inequitable care, and further exacerbating underrepresentation or misrepresentation of certain population groups. Most professionals agreed that patients would feel betrayed if their data were used for profit without their consent, as highlighted by a family doctor. Transparency in commercial practices and strict boundaries on data usage were universally seen as essential to prevent misuse by insurance entities or pharmaceutical industry, because they might promote certain treatments or coverage based on a financial bottom line.

Among insurance providers, the commodification of health data was seen as a double-edged sword. On one hand, it could enhance risk assessments and pricing models; on the other, it raises ethical and regulatory challenges. An expert provided insights on how this commercialisation would allow insurers to refine risk models and offer personalized premiums. However, they also warned against exploitation as the economic asset value of health data is widely acknowledged but a clear emphasis on ethical boundaries must be laid out.

### 3.3 Perception on AI and good health and well-being

The study also explored the perceptions on the utilization of AI in healthcare and whether these outweigh the risks of data commodification. Among the study participants, 12 (28.57%) agreed and 2 (4.77%) strongly agreed; however, interestingly similar proportions were found among those who disagreed or strongly disagreed; whereas 14 (33.33%) maintained a neutral stance. This shows a high level of scepticism and uncertainty among the study participants.

During the qualitative interviews, AI’s role in advancing healthcare and medical research was acknowledged, though accompanied by reservations about safety and equity. Several participants expressed optimism about the potential for AI to improve outcomes, benefit research and also lead to advancements in medical technologies, provided proper oversight was in place: “*If the process includes oversight from independent regulators.*” However, others raised concerns about errors in AI systems, such as “*I fear that errors in AI algorithms could lead to wrong medical decisions.*” The need for AI to prioritize patient-specific needs over generalized trends was frequently mentioned. One participant observed, “*Quality might suffer if decisions are based more on data trends than individual needs.*” This underscores the necessity of striking a balance between technological efficiency and personalized care.

Participants highlighted risks associated with insufficient safeguards and the potential for increased inequality. Smaller healthcare providers were seen as particularly vulnerable in AI-driven ecosystems. Regulatory gaps were another common worry: “*I think the speed of commodification might outpace necessary regulations.*” Perspectives from healthcare professionals revealed a promise in the positively evolving nexus of healthcare and AI with the potential of AI towards advancement in diagnostic precision, but to be wary about the biases and errors in AI-powered tools, which could compromise patient safety. Further, a critical care nurse feared that an over-reliance on algorithms might depersonalize care, reducing the comfort and compassion offered by healthcare professionals especially in cases demanding critical care. They emphasized that healthcare providers must educate patients about the benefits and risks of AI systems to ensure confidence in their implementation.

### 3.4 Putting the patient first

Study participants also provided insights on establishing trust and comfort with the evolving systems and suggested methods that would improve their willingness to share data. These included primarily two methods: development of robust regulatory frameworks and safeguards, and, establishing compensation models for the patients.

Robust regulations were seen as an essential component to protect patient rights and maintain ethical standards. Informed consent and the ability to withdraw participation were recurring themes: “*Patients should be able to withdraw their consent at any time.*” Independent oversight and stringent penalties for misuse were also frequently advocated: “*There should be auditing to ensure compliance with data protection laws.*” Participants stressed the need for transparency and ethical training for entities handling health data. Measures such as encryption, liability frameworks, and patient feedback mechanisms were emphasized. As one participant noted, “*The process must include oversight from independent regulators to ensure accountability and fairness*”.

Further, diverse compensation models were suggested, ranging from direct payments to more innovative approaches: “*Financial compensation might influence my decisions, though even then I would want some sort of assurance regarding security.*” Monetary payments were a common suggestion and participants also mentioned discounts on healthcare services, royalties, and early access to new treatments. Recognition-based models, such as certificates or acknowledgments, were also proposed: “*Patients should receive recognition for their contributions to healthcare advancements.*” Other innovative suggestions included tax incentives, investment credits in healthcare funds, and the option to donate proceeds to charities selected by the patient.

Healthcare professionals also underscored the criticality of robust regulatory frameworks to protect patient privacy, ensure informed consent, and hold entities accountable for data misuse. They advocated for ongoing patient education and transparent communication to build trust and providing patients with options and reaffirming their consent regularly. Administrators recommended making consent forms to be void of jargon, and ensuring data is used solely for patient-centered purposes. Encryption of sensitive data, ethical training for healthcare professionals, and independent oversight were also widely supported.

## 4 Discussion

### 4.1 Main findings

This study attempts to provide a holistic lens with perspectives from patients, healthcare providers, technology experts as well as insurance providers on the commodification of health data, the potential benefits of utilising such datasets and the perceived challenges. Overall, respondents expressed mixed opinions regarding the possible benefits of using AI, whereas most participants agreed on the disadvantages and the challenges. The findings of the study underscore the complex interplay of trust, privacy, and transparency within the nexus of AI and healthcare.

In our study, participants expressed significant concern over the perceived lack of control and transparency in the utilisation of their health data, accompanied by a deep trust deficit in AI systems and algorithms. The underlying distrust provides evidence for an imperative for AI systems developers to include transparent data handling practices and patient-consenting procedures to foster acceptance in Saudi healthcare settings. Similar findings have been reported by Esmaeilzadeh et al. (2021), Khullar et al. (2022), and Kuo et al. (2024) wherein patients raised perceived privacy concerns, lack of trust, potential for bias, as well as limited transparency offered by AI-based systems. Further, there is an overarching fear of reduction of the ‘human’ element in medical care, as various roles that require continual intervention and support by primary caregivers, especially in the management of chronic diseases, may be replaced by AI (Khullar et al., 2022; Kuo et al., 2024; Mittelstadt, 2022). The commodification of health data presents ethical, social and policy challenges that demand robust policy frameworks, and comprehensive and patient-centric solutions. This study’s findings highlight critical areas for policy interventions aimed at building trust, safeguarding patient rights, and fostering equitable frameworks for health data use, with particular attention to Saudi Arabia’s rapidly evolving digital health landscape. This is particularly essential as Saudi Arabia is undergoing significant policy shifts towards a rapid digitalization of healthcare, supported by substantial investments and policy interventions such as the National AI Strategy 2030, which aims to position Saudi Arabia as a leader in AI innovation in healthcare. Comparative studies from Europe and North America show similar worries about data commodification and AI ethics. However, regulatory frameworks and cultural expectations vary. Taking a global perspective could improve the understanding of findings and guide policy changes.

### 4.2 Building trust through patient-centric governance

A fundamental step in addressing concerns on data privacy and lack of control would be to develop governance policies and frameworks that prioritize informed consent, transparency, and accountability. Building on the pillars of Saudi Arabia’s Personal Data Protection Law and National AI Strategy 2030, the policymakers can implement mechanisms assuring informed opt-in consent, repeated scrutiny on the usage of patient data, and transparency in AI-driven healthcare systems. Participants, particularly, patients laid emphasis on the treatment of health data as a personal asset, which is sensitive in nature, and individuals must retain control over its usage. Hence, policymakers must build on existing policy initiatives by implementing comprehensive regulations to ensure patients’ rights are protected. Mandating opt-in consent mechanisms and providing patients to periodically review their data usage intent with the ability to revoke consent at any time would enhance trust in the healthcare system. Those would include stringent encryption of data, effective oversight roles, and defined processes for negatives on patients revoking or changing permanently within allowances for delivery of consent. Advanced encryption techniques, secure access protocols, and anonymization methods are essential to protect sensitive health information. This is particularly essential as (Patsakis and Lykousas, 2023) elaborate how large language models (LLM) can undo document anonymization, using minor knowledge hints to achieve complete deanonymization of data. Therefore, even though the Kingdom of Saudi Arabia Personal Data Protection Law provides stringent provisions to ensure data is deidentified prior to sharing, it is also essential to train researchers, scientists and other practitioners to be proficient in de-identifying the data as per the appropriate standards and guidelines.

Saudi Arabia, leveraging its investment in digital infrastructure and partnerships with global health tech firms, can implement state-of-the-art privacy technologies as a standard. AI algorithms trained on anonymized datasets can provide significant benefits for healthcare delivery without compromising individual privacy. This is exemplified through frameworks laid out in various nations, such as the European Health Data Space. The EHDS has provisions for individuals to opt-out of sharing their personal electronic health data for secondary use, and prohibits the use of this data or identifiable data for the purposes of job offers, advertising or marketing, and activities in conflict with national ethical provisions and laws (Gallese, 2024). While the Kingdom of Saudi Arabia Personal Data Protection Law also lays out these provisions, however, it does not explicitly define the purposes wherein the usage of sensitive data is prohibited, except for the purposes of marketing and to protect patients from biases that may exacerbate inequities among vulnerable populations. Moreover, further clarity is required on its implementation and mechanism for imposition of fines and penalties.

### 4.3 Policies to establish training and awareness protocols

Patients also emphasized on the insufficiency in data usage transparency, decision making, and AI algorithms and health care providers also identified various shortcomings such as hallucinations/incorrect predictions or patterns which may lead to loss of trust among patients. Various studies point towards a low-level of awareness among the medical fraternity on the ethical and moral challenges posed by AI (Al-Ani et al., 2024). Hence, there should be periodic training programs for healthcare practitioners to enhance their understanding of applications of AI, ethics of data, and patient-centered data handling. Therefore, since healthcare providers are expected to serve as a conduit among healthcare technology and the patients, educating them on the use of their data, there is a drastic need to enhance knowledge and awareness among healthcare professionals themselves.

Therefore, it is essential to establish protocols for periodic training of AI tools among healthcare practitioners as well as circulation of awareness materials among patients and other concerned groups on the benefits on AI in diagnostics and treatment, while addressing concerns on errors and potential biases. For instance, the Understanding Patient Data program in the United Kingdom provides resources and materials to clarify how patient data is utilized by the National Health Service in an effort to increase transparency and advocate towards the benefits of AI in healthcare (Understanding Patient Data, n.d.). These efforts can be further supplemented via community engagement efforts to build trust among patients and healthcare providers.

### 4.4 Equitable data sharing and compensation models

The study found universal support and near-unanimous agreement among the patients on increased willingness to permit data usage if compensation is provided. Policymakers in Saudi Arabia must explore policy instruments to enable direct monetary benefits, discounts on healthcare services or tax incentives as potential mechanisms for providing compensation to patients. Policymakers could devise a structured compensation scheme to include such things as a direct payment, a discount on medical services, or tax incentives, and thereby ensure ethical boundaries are not crossed whilst patients stand to gain fairly. However, these mechanisms must be supported by stringent safeguards and regulatory balance to prevent exploitation. By involving patients in the decision-making process regarding how proceeds are used, such as allowing donations to charities or healthcare funds, the system can align economic benefits with broader societal goals. Further, as utilised in other health care technologies, such as vaccines, no-fault, no-liability compensation funds could be obtained by companies that develop these technologies towards insurance that would pay out for an injury or any unforeseen incidents or damages (Thomas, 2017).

### 4.5 Ethical AI integration for patient-centric care

AI’s integration into healthcare must prioritize ethical practices and patient-specific needs. Saudi Arabia’s healthcare sector, supported by its Vision 2030 goals, offers an opportunity to lead in developing ethical AI frameworks that prioritize equity and inclusivity. Regulators should ensure AI algorithms are trained on diverse datasets to avoid biases that could disadvantage minorities or underserved populations. The applicability of commodification of data to improve and enhance healthcare has been put forth by Timmermans and Almeling (2009) wherein they argue for a conceptual readjustment and realignment of commodification such that it might become a driver for health care actions and therefore, it is essential to examine the lens with which AI is integrated into healthcare, so as to treat AI as a tool to implement growth and improvements in healthcare. This may be facilitated through a stringent and incessant monitoring of AI systems to prevent biases, identify and control errors or disproportionate effects on specific groups of people, that could compromise patient safety or reinforce existing inequities. AI developers and healthcare providers should use ongoing audits, inclusive dataset standards, and feedback systems focused on patients. This will help ensure that AI integration is ethical, safe, and fair. Nayak and Walton (2024) also argue for the development of policy and governance mechanisms on the use of AI with safeguards to protect labour, advance human development and foster social welfare.

Furthermore, AI technologies must satisfy regulatory requirements for safety, accuracy and efficacy, with stringent measures for quality control and utilise measures such as k-anonymity and differential privacy to protect data privacy, and foster trust from patients and healthcare providers. Governance protocols must include mechanisms for redressal for individuals or groups that may be adversely affected by decisions based on algorithms. Furthermore, we should put in place clear transparency and accountability measures. This includes regular reporting, algorithm reviews, and easy ways for people to raise concerns. These steps will help build trust and ensure ethical oversight in AI-driven healthcare systems.

AI systems must be integrated into healthcare with responsiveness such that developers and users may continuously provide feedback and assess AI applications with respect to the sustainability of health systems. Regular monitoring and feedback loops should be put in place to adjust AI systems, policies, and operational protocols based on real-world performance and input from stakeholders. This will help maintain safety, effectiveness, and ethical standards.

## 5 Limitations

Our study represents a preliminary investigation into the understanding of perceptions associated with the utilisation of AI in healthcare and the associated health data commodification. While it offers valuable insights, the study is bound by several limitations.

First, the study uses a face-validated questionnaire, rather than one developed through a more rigorous psychometric process, which may limit the validity of the findings in alignment to psychometric tests and measures second, the cross-sectional design restricts the ability to infer changes in perception over time or in response to evolving experiences with AI technologies. Third, the use of convenience sampling introduces a risk of selection bias, potentially limiting the generalizability of the results. This is particularly relevant in the context of Saudi Arabia, where the sampled population may not fully reflect the demographic, cultural, and professional diversity of patients and healthcare providers across different regions and healthcare settings.

Furthermore, the study employed a general framing of AI without distinguishing between specific applications or domains (e.g., diagnostic algorithms, clinical decision support systems, administrative automation). This limits the ability to assess how user perceptions may vary depending on the nature of the AI tool, its level of autonomy, or its proximity to direct clinical decision-making. Additionally, we did not examine how participants’ health status, type of medical condition, or prior exposure to digital technologies may have shaped their views. These factors are likely to influence trust, acceptance, and ethical concerns surrounding AI use in healthcare. Future studies employing more representative sampling strategies, longitudinal designs, and application-specific analyses are needed to generate more actionable insights. The study has several limitations. First, the sample size and scope limit how well we can apply the findings beyond Saudi Arabia. Second, the research did not distinguish between types of AI applications, which may affect perceptions. Third, cultural and legal factors unique to Saudi Arabia may influence patient and stakeholder attitudes; results could vary in other contexts.

## 6 Conclusion

This study provides insights into the perceptions of patients, healthcare providers, technology experts and insurance providers on the commodification of data resultant of AI integration into healthcare service delivery. Currently, there are substantial concerns and challenges regarding data commodification requiring urgent attention and policy intervention. Future research must focus on the development of guidelines on ethical integration of AI and facilitate leveraging on these technologies to improve patient outcomes. Our study highlights the critical need to enhance an in-depth understanding of the factors associated with technology acceptance among a varied group of stakeholders – towards patient-centric care.

The implementation of AI applications in healthcare should be conducted with several necessary considerations. First, it is necessary to develop governance mechanisms that address concerns of data privacy, misuse, trust, communication barriers, transparency of regulatory standards, and liability risks. These governance policies must establish protocols towards data anonymization, enabling opt-in consent that may be revoked at any time, as well as defining the rights and responsibilities of healthcare professionals, developers, and programmers.

Second, it is essential to extend efforts towards training and awareness to enhance AI use in healthcare technologies. These include training to healthcare practitioners on de-identifying datasets, enhancing communication materials addressing patient concerns and fostering opportunities for public discourse and engagement in the development and implementation of these technologies to build trust and ensure inclusivity.

Third, policy instruments must identify modalities to allow compensatory models to enable data-sharing by patients with robust safeguards to prevent exploitation. No-fault, no-liability compensation funds for insurance purposes may allow redressal of any unforeseen incidents and enhance patient trust.

Fourth, regulatory requirements for safety, accuracy and efficacy, with stringent measures for quality control must be met by AI technologies to minimise and gradually eliminate risk of bias or data misuse.

Finally, this study calls for further exploration of diverse perspectives and cross-cultural insights to develop globally relevant frameworks for responsible AI deployment in healthcare, ensuring that innovation aligns with the principles of privacy, equity, and ethical integrity to achieve improvements in healthcare service delivery and patient outcomes.

## Data Availability

The raw data supporting the conclusions of this article will be made available by the author, without undue reservation.
